# Lack of S-RNase-Based Gametophytic Self-Incompatibility in Orchids Suggests That This System Evolved after the Monocot-Eudicot Split

**DOI:** 10.3389/fpls.2017.01106

**Published:** 2017-06-22

**Authors:** Shan-Ce Niu, Jie Huang, Yong-Qiang Zhang, Pei-Xing Li, Guo-Qiang Zhang, Qing Xu, Li-Jun Chen, Jie-Yu Wang, Yi-Bo Luo, Zhong-Jian Liu

**Affiliations:** ^1^State Key Laboratory of Systematic and Evolutionary Botany, Institute of Botany, Chinese Academy of SciencesBeijing, China; ^2^Graduate University of the Chinese Academy of SciencesBeijing, China; ^3^Shenzhen Key Laboratory for Orchid Conservation and Utilization, The National Orchid Conservation Centre of China and The Orchid Conservation and Research Centre of ShenzhenShenzhen, China; ^4^The Centre for Biotechnology and BioMedicine, Graduate School at Shenzhen, Tsinghua UniversityShenzhen, China; ^5^College of Forestry and Landscape Architecture, South China Agricultural UniversityGuangzhou, China; ^6^College of Arts, College of Landscape Architecture, Fujian Agriculture and Forestry UniversityFuzhou, China

**Keywords:** Orchidaceae, self-incompatibility, evolution, transcriptomics and genomics, S-RNase-based GSI

## Abstract

Self-incompatibility (SI) is found in approximately 40% of flowering plant species and at least 100 families. Although orchids belong to the largest angiosperm family, only 10% of orchid species present SI and have gametophytic SI (GSI). Furthermore, a majority (72%) of *Dendrobium* species, which constitute one of the largest Orchidaceae genera, show SI and have GSI. However, nothing is known about the molecular mechanism of GSI. The S-determinants of GSI have been well characterized at the molecular level in Solanaceae, Rosaceae, and Plantaginaceae, which use an S-ribonuclease (S-RNase)-based system. Here, we investigate the hypothesis that Orchidaceae uses a similar S-RNase to those described in Rosaceae, Solanaceae, and Plantaginaceae SI species. In this study, two SI species (*Dendrobium longicornu* and *D. chrysanthum*) were identified using fluorescence microscopy. Then, the S-RNase- and SLF-interacting SKP1-like1 (SSK1)-like genes present in their transcriptomes and the genomes of *Phalaenopsis equestris, D. catenatum, Vanilla shenzhenica*, and *Apostasia shenzhenica* were investigated. Sequence, phylogenetic, and tissue-specific expression analyses revealed that none of the genes identified was an S-determinant, suggesting that Orchidaceae might have a novel SI mechanism. The results also suggested that RNase-based GSI might have evolved after the split of monocotyledons (monocots) and dicotyledons (dicots) but before the split of Asteridae and Rosidae. This is also the first study to investigate S-RNase-based GSI in monocots. However, studies on gene identification, differential expression, and segregation analyses in controlled crosses are needed to further evaluate the genes with high expression levels in GSI tissues.

## Introduction

Orchidaceae, which represents approximately 8% of all vascular plant species and contains five subfamilies (Apostasioideae, Vanilloideae, Cypripedioideae, Orchidoideae, and Epidendroideae), is one of the largest plant families and includes more than 25,000 species that are known for their diverse specialized reproductive and ecological strategies (Givnish et al., 2015). The large morphological variation exhibited by this family is mostly attributable to the striking adaptations these plants have made to attract pollinators, including insects and birds (Barbosa et al., 2009; Holzinger and Pichrtova, 2016). Although the reproductive systems of the members of a given population are important factors in determining their genetic variability, most Orchidaceae are self-compatible (SC), “avoiding” self-pollination by other means (Gontijo et al., 2010). Self-incompatibility (SI) is estimated to occur in 10% of orchid species; however, a majority (72%) of the 61 *Dendrobium* species that are self-pollinated show self-sterility (Johansen, 1990). Interestingly, nearly one-half of orchid SI species are from *Dendrobium*. As one of the largest Orchidaceae genera, the high SI rate in *Dendrobium* species might contribute to their high levels of species diversity.

SI influences seed and fruit setting, the growth of pollen tubes, seed filling, seed germination, and seedling development in Orchidaceae. Therefore, the capsule set, seed filling, and growth of pollen tubes following self- and cross-pollination are the main SI indicators (Millner et al., 2015). Studies performed on Pleurothallidinae and *Dendrobium* (Johansen, 1990; Barbosa et al., 2009; Millner et al., 2015; Pinheiro et al., 2015) suggest that the sites of incompatibility reactions vary among groups, implying that various SI molecular mechanisms exist in Orchidaceae. The pollen tubes of certain *Dendrobium* SI species (e.g., *D. farmeri*) exhibited similar reactions in self- and cross-pollination (Johansen, 1990), while the pollen grains of most self-pollinated flowers, such as *Masdevallia infracta* and *Octomeria, Stelis, Specklinia*, and *Anathallis* species (except *A. microphyta*), did not germinate (Gontijo et al., 2010). In addition, self-pollinations performed across 26 species of *Restrepia* (Orchidaceae) revealed that pollen tubes grew only into the top third of the ovary (Millner et al., 2015). *Pleurothallis adamantinensis* and *P. fabiobarrosii* are strictly self-incompatible because pollen tube growth ceases near the base of the column (Borba et al., 2001).

In gametophytic SI (GSI) systems, the SI phenotype of the pollen is determined by its own (haploid) S genotype, and the pollen tube growth is typically arrested at some point on its path through the transmitting tract towards the ovary (Borba et al., 2001). Although previous studies reported pollen tube reactions of some SI orchid species that were similar to those observed in species with GSI (Borba et al., 2001; Millner et al., 2015), little is known about the physiology and genetic control of SI in Orchidaceae due to their very long lifecycles, which effectively preclude genetic analysis (Johansen, 1990). One type of GSI has been well studied: S-ribonuclease (S-RNase)-based GSI in Rosaceae, Solanaceae, and Plantaginaceae. The female S-determinant is an S-RNase glycoprotein belonging to the RNase-T2 family and with style-specific expression, and the male S-determinant encodes an F-box protein (Yamashita et al., 2004), which has been designated the S-locus F-box (SLF) protein and has pollen-specific expression. According to plant RNase-T2 family phylogenetic analysis, this family can be divided into two different subfamilies: S-RNases involved in the rejection of self-pollen during the establishment of SI in the Rosaceae, Solanaceae, and Plantaginaceae plant families and S-RNase-like RNases-T2. Furthermore, S-RNase-based GSI evolved only once—before the split of Asteridae and Rosidae approximately 120 million years ago (MYA)—which may suggest that other plant families featured this GSI molecular mechanism (Igic and Kohn, 2001; Steinbachs and Holsinger, 2002; Vieira et al., 2008a; MacIntosh et al., 2010). However, the phylogenetic origin of the S-RNase-based SI system remains undated, and thus, whether the same GSI molecular mechanism exists in monocots is unknown.

Amino acid pattern analysis of RNase-T2 genes revealed four patterns: amino acid patterns 1, 2, 3 and 4 (Vieira et al., 2008a). The amino acid motifs encoded by S-RNases differ from those of other RNase-T2 genes (Vieira et al., 2008a; Nowak et al., 2011). Although amino acid patterns 1 and 2 are exclusively found in the proteins encoded by S-RNase lineage genes, amino acid pattern 4 is not found in any protein encoded by these genes (Vieira et al., 2008a; Nowak et al., 2011). This difference can be used to identify putative S-RNase lineage genes. In addition, all S-RNases have an isoelectric point (IP) between 8 and 10 (Roalson, 2003), which may be further refined in the different homologs. The number of introns can also be used to select S-RNase lineage genes because S-RNases have only one or two introns, and these genes are expected to be specifically and highly expressed in pistils, although they can show lower expression levels in stigma and styles. Moreover, S-RNase lineage genes must be demonstrated to have high polymorphism levels, be positively selected, and, in controlled crosses, co-segregate with S-locus alleles. Finally, the phylogenetic position of S-lineage gene homologs and a set of reference genes should be used to determine whether the genes belong to the S-RNase lineage. One male S-determinant gene has been identified in *Prunus* spp. (Rosaceae; the S haplotype-specific F-box (SFB) gene (Ushijima, 2003; Ikeda et al., 2004; Sonneveld et al., 2005; Nunes et al., 2006; Vieira et al., 2008b)), whereas multiple genes have been identified in Pyrinae (Rosaceae; S-locus F-box brothers [SFBB] genes [Cheng et al., 2006; Kakui et al., 2007; Sassa et al., 2007; Minamikawa et al., 2010; De Franceschi et al., 2011; Kakui et al., 2011; Okada et al., 2011; Aguiar et al., 2013]) and Solanaceae [S-locus F-box (SLF) genes (Wheeler and Newbigin, 2007; Kubo et al., 2010; Williams et al., 2014)]. These genes belong to a large gene family, but no typical protein features have been reported to date. The only known feature is pollen-specific expression, which makes identifying pollen S-gene(s) using sequence data alone difficult.

In addition, as the diagnostic marker for the presence or absence of RNase-based GSI, the SLF-interacting S-phase kinase-associated protein 1-like (SKP1-like)1 (SSK1) proteins, have been described only in the SI reactions of Rosaceae, Solanaceae, and Plantaginaceae (Hua and Kao, 2006; Huang et al., 2006; Zhao et al., 2010; Xu et al., 2013). SKP1-like proteins are also adapters that connect several F-box proteins to the SCF complex and are necessary in a wide range of cellular processes involving proteasome degradation (Huang et al., 2006). They are also highly conserved and have a unique C-terminus that comprises 5–9 amino acid residues following the conventional “WAFE” motif that is found in most plant SKP1 proteins (Zhao et al., 2010) and can be easily identified. The sequence “GVDED” is conserved in Rosaceae, and although it is not as well conserved in Solanaceae and Plantaginaceae, the D residue is always found at the last position of the motif. These genes are expressed only in the pollen of Solanaceae, Plantaginaceae, and Pyrinae and the styles of *Prunus* spp. (Hua and Kao, 2006; Huang et al., 2006; Zhao et al., 2010; Xu et al., 2013).

The present study aimed to verify whether the SI molecular mechanism of Orchidaceae is similar to that of S-RNase-based GSI and to further explore the phylogenetic origin of S-RNase-based GSI. Based on the genomic data associated with the representative phylogenetic positions and self-compatibility of four species belonging to three different Orchidaceae subfamilies—*Phalaenopsis equestris* (SC) (Cai et al., 2015), *D. catenatum* (partial SI) (Zhang et al., 2016), *Vanilla shenzhenica* (SC) (Liu et al., unpublished), and *Apostasia shenzhenica* (SC) (Liu et al., unpublished)—and the transcriptome data obtained here for two SI species (*D. longicornu* and *D. chrysanthum*), S-RNase lineage genes and SKP1-like genes were characterized. The two SI species were identified by observing the growth of their pollen tubes after self- and cross-pollination using fluorescence microscopy. Combining amino acid pattern, tissue-specific expression, and phylogenetic analyses, homologous S-RNases and SKP1-like genes were investigated in the four genomes and the transcriptomes of two orchid species. No evidence of RNase-based GSI was found in *D. longicorn* or *D. chrysanthum*, and no S-determinant orthologous sequences were found in the potential S-locus region in four orchid genomes. The results revealed that Orchidaceae GSI might not be determined by S-RNase-lineage genes, as it is in Rosaceae, Solanaceae, and Plantaginaceae. Therefore, we propose that RNase-based GSI might have originated after the split of monocots and eudicots but before the split of Asteridae and Rosidae.

## Materials and Methods

### Identification of SI

Two days after flowering, the plants were self- and cross-pollinated. *D. chrysanthum* pistils were collected 12, 24, 48, 72, and 96 h after self- and cross-pollination (HASP and HACP, respectively), and *D. longicornu* pistils were collected 2, 3, 4, 5, 7, and 9 days after self- and cross-pollination (DASP and DACP, respectively). The pistils were immersed in a fixing solution (formalin-acetic acid-80% alcohol [1:1:8]) for at least 24 h, rinsed in 70% alcohol, softened in a strong (8 N) sodium hydroxide solution for 3 h, and cleared in distilled water. The pistils were stained with 0.1% water-soluble aniline blue dye dissolved in 0.1 N K_3_PO_4_ for 12 h and then observed under a fluorescence microscope (Axioskop 40, Zeiss, Germany). The ultraviolet light used (wavelength: approximately 356 μm) facilitated the examination of the growth of pollen tubes on the style because the pollen tubes were lined with callose, which fluoresced bright yellow-green and contrasted strongly with the bluish or grayish fluorescence of the style and ovary tissues. The lengths of the majority of the pollen tubes in compatible and incompatible styles at specific times after pollination were measured using fluorescence microscopy software. Statistical analyses were performed using the software package GraphPad Prism 6 for Mac OS X (version 6.0c; GraphPad Software, Inc., La Jolla, CA, United States). All values were reported as the mean and SEM. The data were presented as the average of three independent measurements with error bars (SEM) indicated. The data were analysed by two-way repeated measures analysis of variance (ANOVA) using Sidak’s *post hoc* test (^∗∗∗^*P* < 0.001, ^∗∗∗∗^*P* < 0.0001).

### Plant Materials, RNA Extraction, Library Construction, and Sequencing

Mature pollen, styles (containing stigma) and leaves of *D. chrysanthum* and the 3 DACP styles (containing pollen), 3 DASP styles (containing pollen) and leaves of *D. longicornu* were simultaneously collected during the pollination of the same plants, placed in 2-ml tubes in liquid nitrogen, and stored at –80°C until RNA extraction. Three biological replicates of each *D. chrysanthum* tissue were collected.

Total RNA was extracted from *Dendrobium* spp. tissues using an RNA prep Pure Plant Kit, and genomic DNA contamination was removed using RNase-free DNase I (both from Tiangen, Beijing, China). The integrity of the RNA was evaluated on a 1.0% agarose gel stained with ethidium bromide (EB), and its quality and quantity were assessed using a NanoPhotometer^®^ spectrophotometer (IMPLEN, Westlake, CA, United States) and an Agilent 2100 Bioanalyzer (Agilent Technologies, Santa Clara, CA, United States). Because the RNA integrity number (RIN) was greater than 7.0 for all samples, the samples were used for cDNA library construction and Illumina^®^ sequencing, which was completed by Beijing Novogene Bioinformatics Technology Co., Ltd. (China). The cDNA library was constructed using the NEBNext^®^ Ultra^TM^ RNA Library Prep Kit for Illumina^®^ (NEB, United States) with 3 μg of RNA per sample, following the manufacturer’s recommendations. The polymerase chain reaction (PCR) products obtained were purified (AMPure XP system), and the library quality was assessed on the Agilent Bioanalyzer 2100 system. Library preparations were sequenced on an Illumina^®^ HiSeq 2000 platform, generating 100-bp paired-end reads. Raw sequence reads were deposited in the National Center for Biotechnology Information (NCBI) Sequence Read Archive^1^ (SRA) under accession number SRP097204.

Before assembly, adaptor sequences were removed from the raw reads, and FASTQC^2^ reports were generated. Based on this information, the reads were trimmed at both ends. Nucleotide positions with a QC score lower than 20 were masked (replaced by an N), and the resulting high-quality reads were *de novo* assembled and annotated with TRINITY (Haas et al., 2013). The commands and parameters used for running TRINITY were as follows: Trinity –seqType fq –JM 200G –left sample_1.fq –right sample_2.fq –normalize_by_read_set –CPU 32 –output –min_kmer_cov 2 –full_cleanup. The transcript abundance level was normalized using the fragments per kilobase per million reads (FPKM) method, and FPKM values were computed as proposed by Mortazavi et al. (2008). In genes with more than one transcript, the longest was used to calculate the transcript abundance and coverage.

### Homolog Identification and Comparative Analysis

We first collected S-RNase-related RNase-T2 protein sequences from previous studies (Aguiar et al., 2015; Kubo et al., 2015). Then, we combined the genomic data of *P. equestris* (SC) (Cai et al., 2015), *D. catenatum* (partial SI) (Zhang et al., 2016), *V. shenzhenica* (SC) (Liu et al., unpublished), and *A. shenzhenica* (SC) (Liu et al., unpublished) with six transcriptome sequences in the proteome datasets.

These S-RNase-related RNase-T2 protein sequences were used as seeds to search against proteome datasets using basic local alignment search tools (tBLASTn and BLASTp) with default parameters. To confirm the identities of orchid S-RNase-related RNase-T2 candidate genes, the hidden Markov model (HMM)-based HMMER program 3.1b2 (Eddy, 2011) was used to identify all proteins containing a Ribonuclease_T2 domain. This domain (PF00445.16 in the Pfam database) (Finn et al., 2016) was then used to perform local searches in the proteome datasets. Sequences obtained from both methods were then aligned and manually adjusted in Multiple Alignment using Fast Fourier Transform (MAFFT) (Katoh and Standley, 2013) using the E-INS-I alignment strategy for sequence integrity analysis. Sequences with obvious errors were excluded from subsequent analyses. Each predicted sequence was subsequently verified using BLAST searches against public databases, including NCBI, Pfam (Finn et al., 2016) and Simple Modular Architecture Research Tool (SMART) (Letunic et al., 2012), to confirm its reliability. The HMM profiles of the Skp1 gene family (PF01466.17 in the Pfam database) obtained in the analysis were used in local searches. To maximize confidence, putative Skp1 sequences were also aligned and manually adjusted in MAFFT using the E-INS-I strategy for sequence integrity analysis. The IPs of all peptides were calculated in ExPASy (Gasteiger, 2003).

As monocots, most orchids are SC species; therefore, the SSK1 genes may be non-functional or not involved in the SI pathway. Thus, we chose orchid species of several SI degrees and phylogenetic positions and allowed for some variability regarding the “WAFE” and “GVDED” motifs when retrieving sequences. Angiosperms have two types (I and II) of SKP1-like genes, with type II being much longer than type I and encoding chimeric proteins (Kong et al., 2007). Multiple Skp1 homologs from the same species were shown to have evolved at highly heterogeneous rates, indicating that they have different evolutionary histories (Kong et al., 2004, 2007). Due to the effect of long-branch attraction, when both gene types are included, the analysis typically gives unstable results; in addition, SSK1-like genes belong to type I SKP1-like genes (Kong et al., 2004, 2007; Huang et al., 2006; Zhao et al., 2010; Matsumoto et al., 2012; Xu et al., 2013). For these two reasons, type II genes were excluded from the analysis.

### Multiple Sequence Alignment and Phylogenetic Tree Construction

The amino acid sequences of SSK1 genes, SSK1-like genes from selected land plant species (Kong et al., 2004, 2007; Huang et al., 2006; Zhao et al., 2010; Matsumoto et al., 2012; Xu et al., 2013), and Orchidaceae SSK1-like genes were used for phylogenetic analysis. Multiple sequence alignment was carried out using MAFFT with the E-INS-I strategy and adjusted manually as necessary, and the phylogenetic tree was generated by the maximum likelihood method using PhyML 3.0 (Guindon et al., 2010). The approximate likelihood-ratio test (aLRT) branch support, which was based on a Shimodaira-Hasegawa-like procedure, was estimated with a Whelan and Goldman (WAG) model.

The amino acid sequences of Orchidaceae S-RNase like genes and S-RNase-related RNase-T2s from species with S-RNase-based SI, maize and barley (Igic and Kohn, 2001; Hillwig et al., 2010; Kubo et al., 2015), were used for phylogenetic analysis the same method as that described for SSK1-related genes.

The data sets (the alignments file and the tree file of SKP1 and S-RNase protein sequences) are available through TreeBASE^3^, and also in the **Supplementary Files S3, S4**.

## Results

### Pollen tube development

**FIGURE 1:**
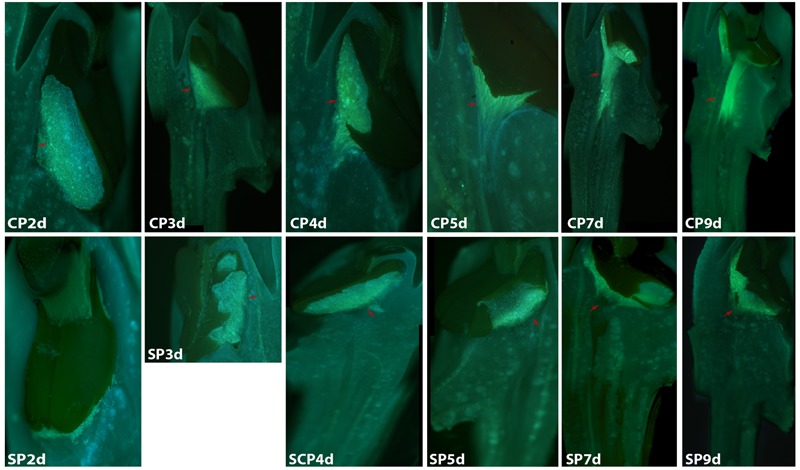
Growth of pollen tubes after the self- and cross-pollination of *Dendrobium longicornu.* CP2d/SP2d: the pollen tubes started to develop in the stigma; CP3d: the pollen tubes started to grow into the style and longer than in SP3d; SP4d, SP5d, SP7d, and SP9d: the self-pollination pollen tubes stopped growing at the top of the style; CP7d: the pollen tubes developed rapidly and reached the ovary. Bar = 100 μm; red arrows indicate the pollen tubes. CP and SP stand for cross-pollination and self-pollination, respectively, d means day.

**FIGURE 2:**
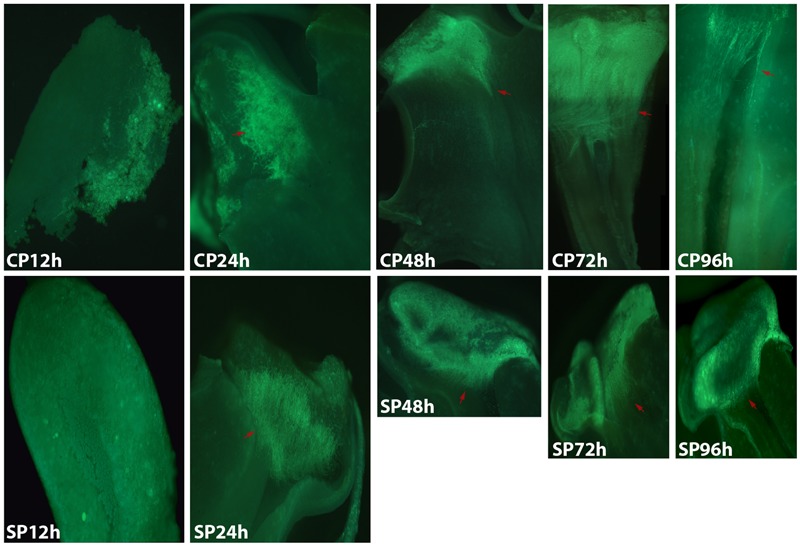
Growth of pollen tubes after the self- and cross-pollination of *D. chrysanthum.* CP12h and SP12h: the pollen grains did not germinate; CP24h/SP24h: the pollen tubes started to develop in the stigma; SP48h, SP72h, and SP96h: the pollen tubes developed slowly or even stopped growing; CP72h and CP96h: the cross-pollination pollen tubes developed rapidly and reached the ovary 72 h after pollination. Bar = 100 μm; red arrows indicate the pollen tubes. CP and SP stand for cross-pollination and self-pollination, respectively, h means hour.

The development of pollen tubes that were stained with water-soluble aniline blue in the pistils of *D. longicornu* and *D. chrysanthum* was observed 12 h after pollination (HAP) using fluorescence microscopy. In *D. longicornu*, pollen tubes started to develop in the stigma 2 DASP or DACP (**Figures 1, 7**). At 3 DAP, when the pollen tubes started to grow into the style, the cross-pollination pollen tubes were longer than the self-pollination pollen tubes; however, approximately 4 DAP, the self-pollination pollen tubes stopped growing at the top of the style. There was a highly significant difference between the self- and cross-pollination pollen tubes beginning at 5 DASP or DACP (**Figure 7**). Approximately 7 DACP, the pollen tubes developed rapidly and reached the ovary. In *D. chrysanthum*, the pollen tubes started to develop in the stigma 24 h after self-pollination or cross-pollination (**Figures 2, 8**). Although the self-pollination pollen tubes grew slowly into the style during the 72 h after pollination and stopped growing before flower (containing ovary) senescence (120 h after pollination), the cross-pollination pollen tubes developed rapidly and reached the ovary 72 h after pollination. There was a highly significant difference between the self- and cross-pollination pollen tubes beginning at 48 HASP or HACP (**Figure 8**).

Overall, the germination of pollen tubes inside the stigma of *D. longicornu* occurred later than that in *D. chrysanthum*, and their growth took longer in *D. longicornu* styles than in *D. chrysanthum* styles. The self-pollination pollen tubes of *D. longicornu* grew to the top of the style before flower senescence, while in *D. chrysanthum*, they stopped growing before flower senescence. Thus, SI was identified in two orchid species by observing the development of pollen tubes using fluorescence microscopy.

### SKP1-Like Genes in Orchidaceae

**FIGURE 3:**
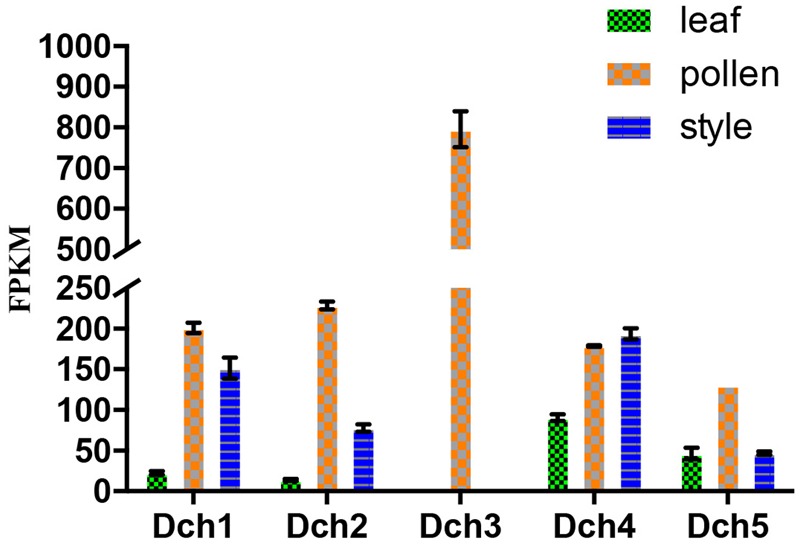
Expression analysis based on the fragments per kilobase per million reads (FPKM) performed for the putative SKP1-like genes in the various tissues (i.e., leaf, pollen, and style) of *Dendrobium chrysanthum* (see also Supplementary Table S1).

We retrieved 42 sequences from orchids, including five *A. shenzhenica* (SC), two *V. shenzhenica* (SC), six *D. catenatum* (partial SI), and five *P. equestris* (SC) genome sequences; four sequences from the leaves and self- and cross-pollinated styles (containing pollen) of *D. longicornu* (SI); and four sequences from the leaves, five sequences from the pollen, and three sequences from the styles (containing stigma) of *D. chrysanthum* (SI). Two genes had a “WAFGE” motif rather than the conventional “WAFE” motif in the 3’ region; that is, a glycine was inserted between phenylalanine and glutamic acid. One of these genes was found in the leaves, pollen, and styles of *D. chrysanthum* (*Dch1_L, Dch1_P*, and *Dch1_S*, respectively) (**Supplementary File S1**), and the other was expressed in the styles (containing stigma and pollen) of *D. longicornu* (*Dlo1_O* and *Dlo1_S*) (**Supplementary File S1**). Similarly, the conserved C-terminus with the “WAFE” motif was replaced by “WAFAE” in one *D. catenatum* sequence (*Dca004172*) (**Supplementary File S1**), and a 16-amino acid deletion was found near the C-terminus of this sequence. Another *D. catenatum* sequence (*Dca007256*) presented a “WAFDLICL” motif, and one *A. shenzhenica* sequence (*Ash003413*) presented a “WAFEPQQ” motif at the C-terminus (**Supplementary File S1**). The *Dca007256* gene was expressed in stigma, and the *Ash003413* gene was also expressed in leaves, stems and tubers (data not shown). The FPKM analysis of the combined transcriptome data of the three *D. chrysanthum* tissues revealed that all of the abovementioned genes were expressed in *D. chrysanthum* leaves and, therefore, were not pollen-specific, except *Dch3_P* (**Figure 3**, **Supplementary File S2**, and Table S1).

In the phylogenetic analysis, SSK1 genes, SSK1-like genes from selected land plant species, and Orchidaceae SSK1-like genes were combined. The SSK1-like genes from Orchidaceae were divided into group I (35 genes) and group II (6 genes), and one gene was clustered with one *A. thaliana* gene (*ASK2*) (**Figure 5**) shown in blue. Within group I, all genes expressed in the style and/or pollen were also expressed in the leaf and thus were not pollen-specific genes (**Supplementary File S2** and Table S1); this was the case for *Dlo2_L, Dlo2_O*, and *Dlo2_S* or *Dch2_L, Dch2_P*, and *Dch2_S*. In group II, which was a sister to that formed by the SSK1 genes, no genes were expressed in leaf tissues, and with a few exceptions, all genes in this group were expressed in the pollen of self- and cross-pollinated orchids, suggesting that these might be pollen-specific genes; the exceptions were *PEQU_01554* and *Ash012058*, which were expressed in the leaf, stem, root, and pollen, and *Dca025410*, which was also found in the root tissue (data not shown).

The phylogenetic tree positions and tissue-specific expression analyses revealed that the genes *Dch3_P* in *D. chrysanthum* and *Dlo3_S* and *Dlo3_O* in *D. longicornu* may be Orchidaceae SSK1-like genes, despite their lack of a “GVDED” motif. Indeed, in SI Orchidaceae, the conserved “WAFE/D” motif might have evolved into a sequence different from that generally observed in S-RNase-based GSI.

### RNase-T2 S-Lineage Genes in Orchidaceae

As RNase-based GSI might be present in Orchidaceae, we attempted to identify an S-RNase gene in this family. Based on four criteria, S-RNase-like genes were identified in *A. shenzhenica, V. shenzhenica, D. catenatum*, and *P. equestris* genomes and the transcriptomes from the leaves, pollen, and styles of *D. chrysanthum* and the leaves and self- and cross-pollination styles of *D. longicornu*. The criteria were as follows: (1) The genes were similar, at the amino acid level, to those involved in S-RNase-based GSI in Rosaceae, Solanaceae, and Plantaginaceae. (2) The genes encoded a protein in which amino acid pattern 4 was absent; this pattern is found in proteins encoded by non-S-RNase lineage genes only (Vieira et al., 2008a; Nowak et al., 2011). (3) The genes encoded a protein with an IP higher than 7.5 because S-RNases are always basic proteins (Igic and Kohn, 2001; Roalson, 2003). Finally, (4) the genes should be mainly expressed in the style. Given that little is known about the physiology and genetic control of SI in Orchidaceae, we chose SC species (*A. shenzhenica, V. shenzhenica*, and *P. equestris*), a partial SI species (*D. catenatum*), and SI species (*D. chrysanthum* and *D. longicornu*) to identify putative S-genes, although mutations that disrupt the coding region might exist in the putative S-locus region (Tao et al., 2007).

We identified 34 RNase-T2-like genes in Orchidaceae: five in *A. shenzhenica* (SC); four in *V. shenzhenica* (SC); three in *D. catenatum* (partial SI); five in *P. equestris* (SC); two in the leaves, three in the self-pollination styles, and three in the cross-pollination styles (containing pollen) of *D. longicornu* (SI); and three in the leaves, three in the pollen, and three in the styles (containing stigma) of *D. chrysanthum* (SI). The features of all 34 gene sequences, including the IP, sequence motifs, intron numbers, and gene location, are summarized in **Table 1**. The gene intron numbers in *D. longicornu* and *D. chrysanthum* were determined by an alignment with *D. catenatum* and *P. equestris* genes. Six genes had IPs higher than 7.5: *Ash010024, Ash010025, Dlo9_L, Dlo9_S, Dch6_S*, and *VN_GLEAN_10017549*. Twenty-five genes presented amino acid pattern 4 ([R]) and, thus, had S-like amino acid patterns rather than S-RNase amino acid patterns. The patterns “CGS” and “CSS” were found in eight and one gene sequence, respectively. Among the eight “CGS” genes, *Dlo9_L, Dlo9_O*, and *Dlo9_S* were the same gene expressed in different *D. longicornu* tissues, suggesting that this gene was not involved in RNase-based GSI because no style-specific expression was observed. The genes *Dch8_L, Dch8_P*, and *Dch8_S* in *D. chrysanthum* were also the same gene, demonstrating that this gene is also not involved in RNase-based GSI processing. The other two genes (*PEQU_20427* and *VN_GLEAN_10005747*) were found in the SC species *P. equestris* and *V. shenzhenica*; we investigated the functions of the genes located at the two gene scaffolds and analyzed the expression levels of the genes with SLF function (data not shown). The results indicated that these genes were not at the putative S-locus region and not primarily expressed in the style, which also suggested that they are not involved in RNase-based GSI processing. The gene *Ash013339*, which contained the sequence “CSS” in the SC species *A. shenzhenica*, was also determined to not be involved in RNase-based GSI based on the results of adjacent gene function and expression analyses (data not shown).

**Table 1:** The RNases-T2 found in *Apostasia shenzhenica, Vanilla shenzhenica, Phalaenopsis equestris*, and *Dendrobium catenatum* genomes, and in *D. chrysanthum* and *D. longicornu* transcriptomes according to Vieira et al. (2008a).

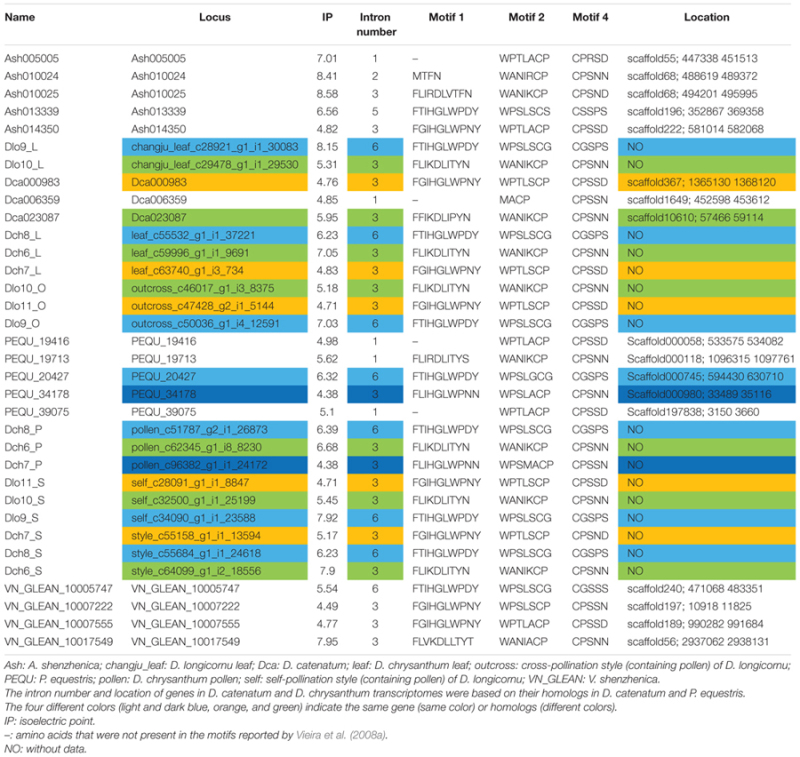

**FIGURE 4:**
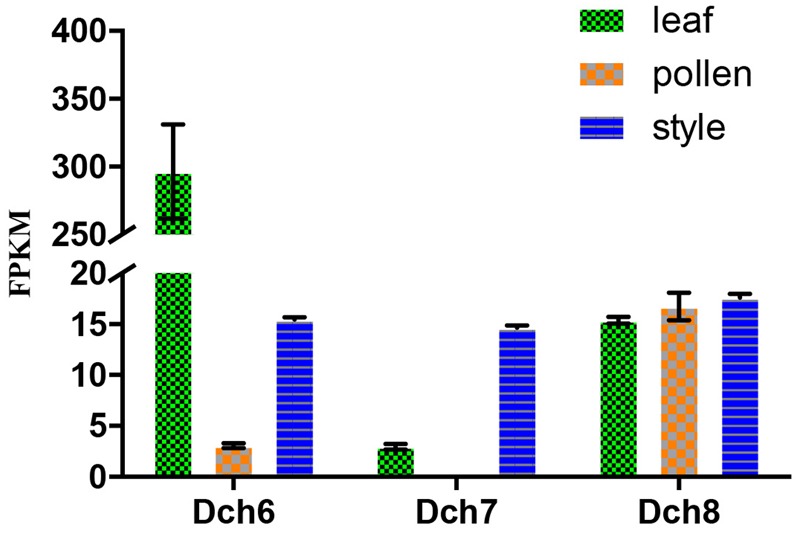
Expression analysis based on the fragments per kilobase per million reads (FPKM) performed for the putative S-RNase-like genes in the various tissues (i.e., leaf, pollen, and style) of *Dendrobium chrysanthum* (see also Supplementary Table S2).

Phylogenetic analyses of these 34 genes and S-RNase-related RNase-T2s from species with S-RNase-based SI, maize and barley, were performed (**Figure 6**). The classification of RNase-T2s into Class-I RNase-T2s, Class-II RNase-T2s, and the S-RNase clade followed that of previous studies (Igic and Kohn, 2001; Hillwig et al., 2010; Kubo et al., 2015). The 34 genes were clustered into Class-I RNase-T2s or Class-II RNase-T2s and were therefore not involved in RNase-based GSI. Three orchid clusters were found in Class-I RNase-T2s: Class-I orchid RNase-T2s I; Class-I orchid RNase-T2s II; and Class-I orchid RNase-T2s III. The genes in Class-I orchid RNase-T2s I clustered with *Zea mays kin1* (AAB37265.1), which is an S-RNase-like gene, and with *Hordeum vulgare* subsp. *vulgare rsh1* (AAF45043.1), which is exclusively expressed in young leaf tissues. The genes within Class-I orchid RNase-T2s II and Class-I orchid RNase-T2s III grouped with *Nicotiana alata* RNase *NE* (AAA21135.1), which is not linked to the SI locus and not specifically expressed in styles, and *Prunus dulcis* RNase *PD2* (AAG31930.1), which is predominantly expressed in petals, the pistils of open flowers, and leaves. Class-II orchid RNase-T2s were a sister group to *Solanum lycopersicum* RNase *LER*, which is not specifically expressed in styles (Kothke and Kock, 2011); *Antirrhinum majus* × *Antirrhinum hispanicum AhSL28* (an S-RNase-like gene), which is not only expressed in pistils; and *Nicotiana glutinosa* RNase *NGR2*, which is constitutively expressed in leaves. Overall, the orchid S-RNase-like genes clustered with S-RNase-like genes that were not specifically expressed in styles (**Figure 4, Supplementary File S2**, and Table S2), suggesting that these genes might not be involved in orchid SI.

**FIGURE 5:**
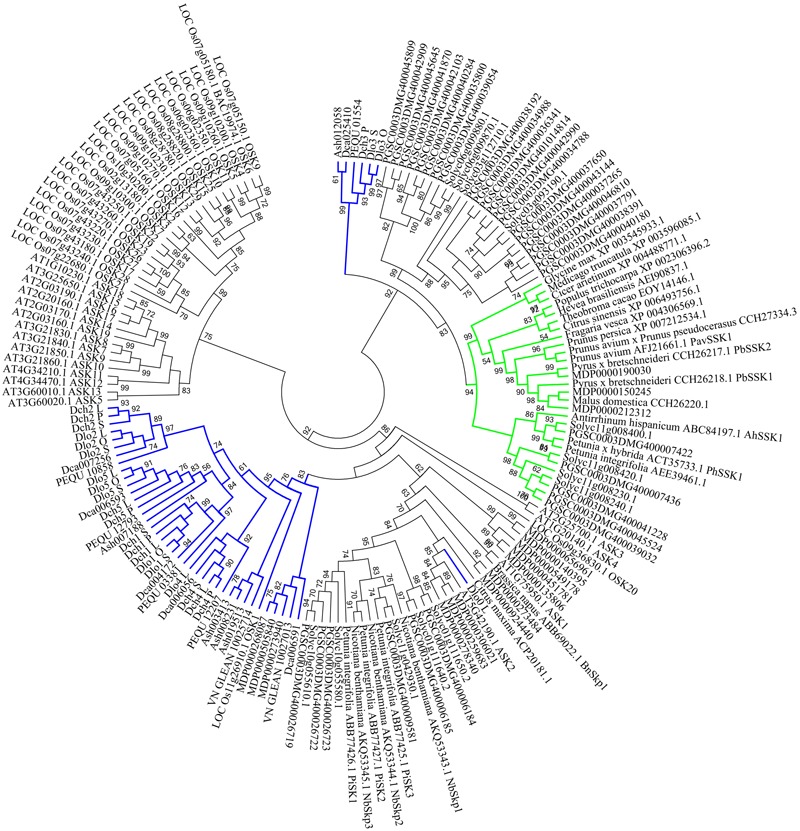
Unrooted maximum likelihood phylogenetic tree based on the aligned amino acid sequences of 42 Skp1-like orchid proteins and 129 Skp1-like proteins from other plants. The 129 deduced amino acid sequences are from *Antirrhinum hispanicum* (AhSSK1), *Arabidopsis thaliana* (ASK1-ASK19, except ASK6), *Brassica napus* (BnSkp1), *Cicer arietinum* (XP_004488771.1), *Citrus maxima* (ACP20181.1), *Citrus sinensis* (XP_006493756.1), *Fragaria vesca* (XP_004306569.1), *Glycine max* (XP_003545933.1), *Hevea brasiliensis* (AEI90837.1), *Oryza sativa* (25 Skp1-like proteins), *Malus domestica* (CCH26220.1 and 16 Skp1-like proteins), *Medicago truncatula* (XP_003596085.1), *Nicotiana benthamiana* (NbSkp1- NbSkp3), *Petunia* spp. (AEE39461.1, PiSK1-PiSK3 and PhSSK1), *Solanum tuberosum* (32 Skp1-like proteins), *Prunus* spp. (PavSSK1, CCH27334.3, XP_007212534.1), *Pyrus* sp. (PbSSK1 and PbSSK2), *Theobroma cacao* (EOY14146.1), *Solanum lycopersicum* (13 Skp1-like proteins), and *Populus trichocarpa* (XP_002306396.2). The SSK1 family is highlighted in green, and SKP1-like genes are highlighted in blue. Values on branches are bootstrap values from SH-like amino acid analysis.

**FIGURE 6:**
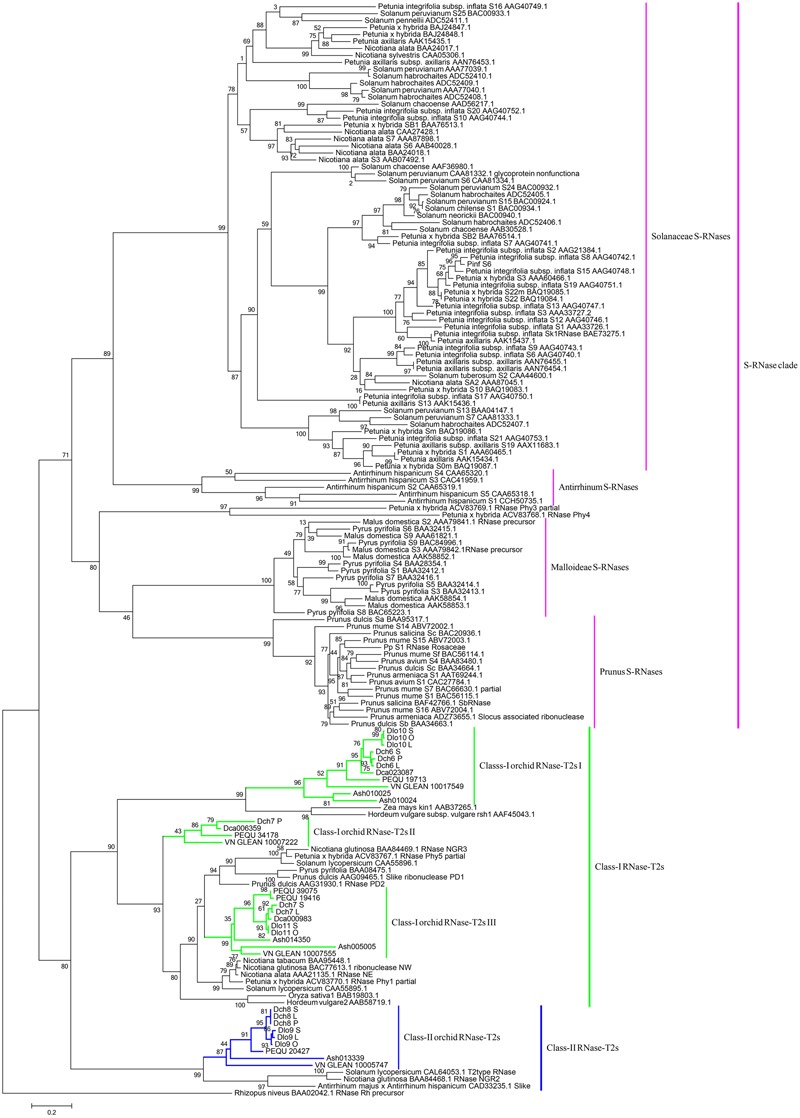
Maximum likelihood phylogenetic tree of the RNase-T2-like genes obtained from orchid species and S-RNase-related RNase-T2s obtained from species possessing S-RNase-based SI, maize, and barley. The tree was constructed in PhyML 3.0 and is based on S-RNase-related RNase-T2s from Solanaceae, Plantaginaceae, and Rosaceae and their homologs in four orchid genomes, two SI *Dendrobium* species transcriptomes, maize, and barley. The classification of RNase-T2s was based on previous studies. RNase-T2s from the filamentous fungus *Rhizopus niveus* were used as the outgroup. Gene subgroups are indicated with different colors. Taxon labels are depicted in pink for the S-RNase clade, which contains Solanaceae S-RNases, *Antirrhinum* S-RNases, Maloideae S-RNases, and *Prunus* S-RNases; in green for Class-I RNase-T2s, which include Class-I orchid RNase-T2s I, Class-I orchid RNase-T2s II, and Class-I orchid RNase-T2s III; and in blue for Class-II RNase-T2s, which contain Class-II orchid RNase-T2s. Values on branches are bootstrap values from SH-like amino acid analysis.

## Discussion

Phylogenetic analysis based on the RNase-T2 genes from four Orchidaceae genomes; the pollen, style, and leaf transcriptomes of *D. chrysanthum*; and the pollen, self-pollination style, and cross-pollination style (containing pollen) transcriptomes of *D. longicornu*, which represent SC, partial SI, and SI species, respectively, suggested that these genes were not phylogenetically related to S-RNases and were clustered with Class-I and Class-II RNase-T2s from other species. It could be argued that S-RNases were not expressed in the selected tissue transcriptomes of the two SI species and that the S-locus region might not be present in the four available genomes. If the SI molecular mechanisms were similar to S-RNase-based GSI, the S-locus region should be present in SC orchids, although male and female S-determinant genes might be truncated and/or non-functional. In Rosaceae, SC species are present in the S-locus region, but the S-RNase and SFB genes are non-functional (Tao et al., 2007); a similar pattern has been described in Brassicaceae SI systems. For instance, although the S-locus is present in the genome of SC *Arabidopsis thaliana*, the genes determining S-specificity are non-functional (Bechsgaard et al., 2006; Boggs et al., 2009). Moreover, the open reading frames (ORFs) of all S-locus cysteine-rich (SCR) alleles and some S-receptor kinase (SRK) alleles are truncated in *Capsella rubella* (Guo et al., 2009), and one *A. lyrata* haplotype, Aly-S38, which is very similar to *C. rubella*, contains a closely related SCR with a truncated ORF and an SRK with a complete ORF (Guo et al., 2011). Thus, the genomes of SC species can also aid in the identification of putative S-locus genes.

Analyses of *D. chrysanthum* and *D. longicornu* S-RNase-like genes revealed no specific gene expression in the style, and amino acid pattern 4 was present in all identified orchid S-RNase-like genes, suggesting that GSI in *D. chrysanthum* and *D. longicornu* is not S-RNase based. We analyzed the expression of the S-RNase-like adjacent genes that were annotated as having SLF function (data not shown), which were also did not exhibit pollen-specific expression patterns, suggesting a non-S-RNase-based GSI mechanism. As S-RNase-based GSI markers, SSK1 genes are typically found in Rosaceae, Solanaceae and Plantaginaceae species. One of the SKP1-like genes identified was specifically expressed in the pollen; this might suggest that the GSI of Orchidaceae is not S-RNase based but does involve SSK1. Nevertheless, to clarify the molecular mechanism of SI in this family, analyses of gene expression, diversity level and segregation in controlled crosses as well as S-locus region identification should be performed to determine which gene(s) are involved in S-pistil specificity.

**FIGURE 7:**
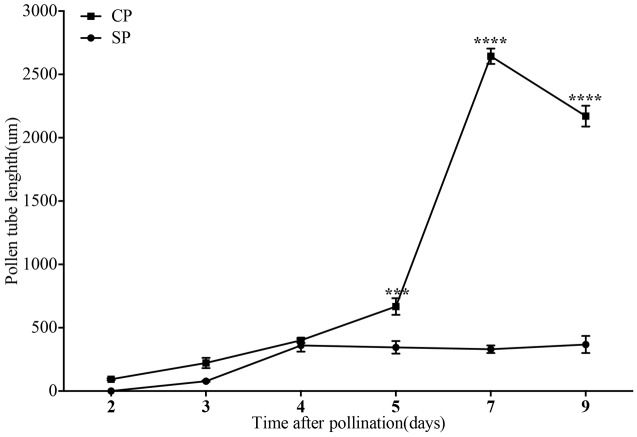
The lengths of the majority of *D. longicornu* pollen tubes present in compatible and incompatible styles at different times after pollination. Pollen tubes were measured at 2 D, 3 D, 4 D, 5 D, 7 D, and 9 D after pollination. Each point is a mean value based on measurements of tubes in three styles, and the error bars represent ± the standard error of the mean. *P*-values were calculated by two-way repeated measures analysis of variance (ANOVA) using Sidak’s *post hoc*-test in GraphPad Prism 6 (^∗∗∗^*P* < 0.001, ^∗∗∗∗^*P* < 0.0001). CP and SP stand for cross-pollination and self-pollination, respectively, D means day.

**FIGURE 8:**
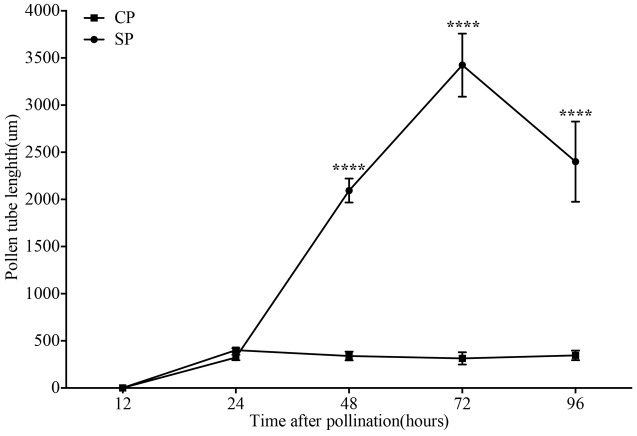
The lengths of the majority of *D. chrysanthum* pollen tubes present in compatible and incompatible styles at different times after pollination. Pollen tubes were measured at 12, 24, 48, 72, and 96 h after pollination. Each point is a mean value based on measurements of tubes in three styles, and the error bars represent ± the standard error of the mean. *P*-values were calculated by two-way repeated measures analysis of variance (ANOVA) using Sidak’s *post hoc* test in GraphPad Prism 6 (^∗∗∗^*P* < 0.001, ^∗∗∗∗^*P* < 0.0001). CP and SP stand for cross-pollination and self-pollination, respectively, h means hour.

The growth of pollen tubes following self- and cross-pollination was observed using fluorescence microscopy and revealed that *D. chrysanthum* and *D. longicornu* are SI species (**Figures 7, 8**). The pollen from both pollination types germinated one and two DAP in *D. chrysanthum* and *D. longicornu*, respectively, but the self-pollination pollen tubes grew much more slowly than the cross-pollination pollen tubes (**Figures 7, 8**). The delay in pollen tube growth may be critical for the SI reaction because by the time the self-pollination pollen tubes penetrate the ovary, the pistil may have already been primed for abscission, as self-pollination flowers fade earlier than normal flowers. After self-pollination, the development of the pollen tube in SI orchids slowed on the stigmatic surface and became arrested at different positions on the pistil that varied among species, similar to the pollen tubes of self- and cross-pollinated *D. farmeri* (Johansen, 1990). The site of the incompatibility reaction in species of representative genera of the main clades of Pleurothallidinae, the largest myophilous group in Orchidaceae, has been investigated (Barbosa et al., 2009); however, most pollen grains of self-pollinated *M. infracta* and *Octomeria, Stelis, Specklinia*, and *Anathallis* (except *A. microphyta*) species failed to germinate. By contrast, in *Acianthera* spp. and *A. microphyta*, pollen tube growth after self-pollination was similar to that observed after cross-pollination until approximately seven days after pollination. However, from that point onward, the self-pollination pollen tubes began to appear abnormal, with irregular trajectories, variations in their diameters, and excessive callose deposition. Approximately 15 DAP in these flowers, pollen tubes with abnormal characteristics had reached the base of the column, although they never penetrated the ovary. In addition, the pollen tubes of *Acianthera saurocephala* never reached the base of the column. Recently, self-pollinations of 26 *Restrepia* species were performed, but pollen tubes grew only into the top third of the ovary (Millner et al., 2015). The variety of incompatibility reaction sites reported suggests that more than one molecular mechanism for SI may exist.

According to the S-RNase-based GSI and SSK1 homologous genes identified and the sites of the incompatibility reaction, the SI molecular mechanism of Orchidaceae might differ from that determined for S-RNase-based GSI, and diverse SI molecular mechanisms may exist. According to the phylogenetic analyses of the RNases-T2, S-RNase-based GSI evolved only once, before the split of Asteridae and Rosidae approximately 120 MYA (Igic and Kohn, 2001; Steinbachs and Holsinger, 2002; Vieira et al., 2008a). However, the phylogenetic origin of the S-RNase-based GSI system remained unresolved. In the present study, homologs of the male and female SI determinants of S-RNase-based GSI were first investigated in Orchidaceae (monocotyledons) using genome and transcriptome data, which suggested that this RNase-based GSI system might have originated after the split of monocots and eudicots but before the split of Asteridae and Rosidae. Further research on other monocotyledons and Orchidaceae species is needed to confirm this hypothesis.

## Author Contributions

S-CN, Z-JL, and Y-BL conceived and designed the study. S-CN and G-QZ prepared the final datasets. S-CN, Y-QZ, J-YW, and QX analyzed and acquired the data. S-CN, JH, and P-XL collected the plant materials. S-CN, Y-BL, and Z-JL wrote the manuscript. All authors read and approved the final manuscript.

## Conflict of Interest Statement

The authors declare that the research was conducted in the absence of any commercial or financial relationships that could be construed as a potential conflict of interest.

## Footnotes

1 http://www.ncbi.nlm.nih.gov/Traces/sra

2 http://www.bioinformatics.babraham.ac.uk/projects/fastqc/

3 http://purl.org/phylo/treebase/phylows/study/TB2:S21138

## Supplementary Material

FILE S1 Multiple alignment of orchid SKP1-like protein sequences.

FILE S2 The FPKM values of SKP1-like genes (Supplementary Table 1) and S-RNase like genes (Supplementary Table 2) in different tissues of *D. chrysanthum*.

FILE S3 The alignment file and the tree file of SKP1 protein sequences.

FILE S4 The alignment file and the tree file of S-RNase protein sequences.
